# HEALTH INTERVIEWING IMPROVES THE MEDICAID HOME AND COMMUNITY-BASED SERVICES HOME CARE AIDE-CLIENT RELATIONSHIP

**DOI:** 10.1093/geroni/igz038.489

**Published:** 2019-11-08

**Authors:** Margaret Danilovich, Rebecca E Johnson, Laura Diaz, Lara Boyken

**Affiliations:** 1 Northwestern University, Chicago, Illinois, United States; 2 Northwestern, Chicago, Illinois, United States

## Abstract

We investigated the feasibility of a Medicaid Home and Community-based Services home care aide (HCA) led health interview with clients (n=21) during usual care services provided by a single provider. We provided interview training in English and Spanish and HCAs (n=21) conducted five interviews using a card sort methodology to elicit client care preferences. The interviews consisted of five topics relative to care: 1) food and drink, 2) physical activity and mobility, 3) self-care, 4) home environment, and 5) how I spend my time. HCAs audio-recorded interviews and photographed card sorts for analysis. We conducted semi-structured interviews by telephone with clients and focus groups with HCAs, to evaluate the health interviewing experience. We transcribed interview recordings and evaluated fidelity to the health interview script. We administered the Your Health Orientation, Willingness to Communicate, and PROMIS-global health to clients and the Active Empathetic Listening Scale to HCAs. We used t-tests to investigate changes in survey outcomes pre and post interviews. Results show it is feasible to train English and Spanish speaking HCAs in a simple, health interviewing technique to elicit care preferences from clients. Doing so contributes new knowledge on client preferences. Clients desire HCAs who provide empathy, compassion, and motivation, and HCAs observed that interviewing clients helped them to better understand their care recipient’s needs. Future work should determine how embedding health communications training as part of orientation to client care, would influence HCA retention rates, as well as modify client health outcomes.

